# Identification of Core Genes of Toll-like Receptor Pathway from *Lymantria dispar* and Induced Expression upon Immune Stimulant

**DOI:** 10.3390/insects12090827

**Published:** 2021-09-14

**Authors:** Long Liu, Yu-Shan Wei, Dun Wang

**Affiliations:** State Key Laboratory of Crop Stress Biology for Arid Areas, Northwest A&F University, Yangling 712100, China; dragonliulong@foxmail.com (L.L.); weiyushan123@foxmail.com (Y.-S.W.)

**Keywords:** Toll-like receptor, innate immunity, *Lymantria dispar*, expression regulation, baculovirus infection

## Abstract

**Simple Summary:**

The gypsy moth, *Lymantria dispar* is one of the main forest defoliators worldwide. The baculovirus, *Lymantria dispar* multiple nucleopolyhedrovirus (LdMNPV), can naturally control this pest and is safe to non-target organisms. However, Toll-like receptor (TLR) signaling pathway, which plays a critical role in innate immunity both in vertebrates and invertebrates, and its underlying immune mechanism against LdMNPV invasion in *L. dispar* have not been investigated. In this study, eleven TLRs and five downstream TLR pathway components of *L. dispar* were identified. All of these proteins comprised typical domain architecture. Primary and tertiary structure analysis revealed that Toll/interleukin-1 receptor (TIR) domains of TLRs from *L. dispar* were conserved during evolution. Expression analysis showed that several *TLRs* and all identified downstream genes of TLR pathway in *L. dispar* were significantly up-regulated in response to LdMNPV infection, implying that the TLR pathway of *L. dispar* was activated and may be involved in *L. dispar* innate immunity against LdMNPV infection. Taken together, this research contributed to the clarification of innate immunity in *L. dispar*.

**Abstract:**

The gypsy moth, *Lymantria dispar*, is a polyphagous forest pest worldwide. The baculovirus, *Lymantria dispar* multiple nucleopolyhedrovirus (LdMNPV) is a natural pathogen of *L. dispar*. The Toll-like receptors (TLR) pathway plays a crucial role in both innate and adaptive immunity in animals. However, The TLR pathway and its underlying immune mechanism against baculovirus in *L. dispar* have not been explored. In this study, eleven TLRs and five downstream TLR pathway components were identified and characterized from *L. dispar*. Structural analysis indicated that intracellular Toll/interleukin-1 receptor (TIR) domains of LdTLRs and LdMyD88 contained three conserved motifs, and the 3D structures of TIR domains of LdTLRs possessed similar patterns in components arrangement and spatial conformation. The TLR proteins of *L. dispar* were placed into five monophyletic groups based on the phylogenetic analysis. *LdTLR1*, *2*, *5*, *6*, *7*, *8* and all identified downstream TLR pathway factors were highly induced upon LdMNPV infection, indicating that the TLR pathway of *L. dispar* was activated and might play a role in the immune response to LdMNPV infection. Collectively, these results help elucidate the crucial role of the TLR pathway in the immune response of *L. dispar* against LdMNPV, and offer a foundation for further understanding of innate immunity of the pest.

## 1. Introduction

For multicellular animals, innate immunity is an efficient first line of defense to avoid pathogen invasion and activate different signaling pathways [1]. Invertebrates, such as insects, lack an adaptive immune system and mainly depend on the innate immune system to counter the diverse pathogens that include fungi, bacteria and viruses [2]. The innate immune system is characterized by the activation of various pattern recognition receptors (PRRs) responsible for recognizing various pathogen-associated molecular pattern (PAMP), molecules present in the pathogen but not found in the host [3]. There are three signaling pathways in insect immune responses, including the Toll (or Toll-like receptor, TLR) pathway, immune deficiency (Imd) pathway and Janus kinase signal transducer and activator of transcription (JAK/STAT) pathway [4]. The Toll/TLR pathway plays an important role both in insect development and immunity, whereas the Imd pathway functions exclusively in immunity [5]. The JAK/STAT pathway is multi-functional, controlling not only immune-regulatory functions but also other biological activities such as cell proliferation, differentiation and apoptosis functions [6].

The Toll protein in *Drosophila melanogaster* was first identified, then its orthologs in mammals and other organisms were named TLRs. TLRs have been characterized in a large number of vertebrates and invertebrates [7]. For instance, 10 and 12 functional TLRs have been found in humans and mice separately. TLRs are a class of PRRs, which are characterized by an extracellular leucine-rich repeat (LRR) region, a transmembrane domain and an intracellular Toll/interleukin-1 receptor (TIR) domain [8]. The extracellular LRR region contributes to pathogens recognition, while the intracellular TIR domain activates the downstream signal transduction via TIR-domain containing adaptors, such as MyD88 (Myeloid differentiation factor 88) or TIRF (TIR-domain-containing adapter-inducing interferon-β) [9]. The Toll-like signaling pathway is comprised of at least two distinct pathways, MyD88-dependent pathway and MyD88-independent pathway. The MyD88-dependent pathway is involved in the activation of nuclear factor kappa B (NF-κB) and mitogen-activated protein kinase (MAPK) pathways by recruiting and activating IL-1 receptor-associated kinase (IRAK), and contribute to the expression of pro-inflammatory cytokines such as tumour necrosis factor-α (TNF-α). The MyD88-independent pathway is linked to the induction of type I interferons (IFNs) to increase the expression of interferon-inducible genes [10].

Although the role of the TLR pathway in bacterial and fungal infection has been well demonstrated, accumulated evidences revealed that it is also important for antiviral response in invertebrates [11]. Toll pathway effectors play an important role in countering Vesicular Stomatitis virus (VSV) and *Drosophila* X virus (DXV) infection in *Drosophila* [11,12] and Dengue virus (DENV) infection in *Aedes mosquito* [13]. However, there are few studies to investigate the role of the TLR pathway in mediating resistance to baculovirus, which is a large DNA virus and an important biology factor of naturally controlling insect populations. The gypsy moth, *Lymantria dispar* (Lepidoptera: Lymantriidae), is a polyphagous insect pest of forests worldwide, whose larvae have been reported to exploit a wide variety of host plants belonging to 400–600 species [14]. The TLR pathway and its underlying immune mechanism against baculovirus in *L. dispar* have not been comprehensively addressed to date. Studies on the key factors of the innate immune system can provide valuable information for new strategies for the integrated management of this pest.

In the present work, several core components from the TLR signaling pathway, including eleven TLRs and five downstream molecules (MyD88, IRAK1, IRAK4, JNK and AP-1), were identified and functionally characterized for the first time from *L. dispar* by the analysis of domain composition, primary and tertiary structure and phylogeny. Furthermore, in order to explore their potential antiviral function against baculovirus, the transcriptional levels of these core genes were determined under immune stimulation by *Lymantria dispar* multiple nucleopolyhedrovirus (LdMNPV) which is a baculovirus specifically infecting *L. dispar* larvae. Results revealed that several *TLRs* and all identified downstream genes of the *L. dispar* TLR pathway were highly induced indicating that the TLR pathway may be involved in the immune reponse against LdMNPV infection. Taken together, our study of core genes of the TLR signaling pathway in *L. dispar* would deepen our understanding of insect innate immunity and the internal mechanisms of the insect TLR family responding to the invasion of viral pathogens.

## 2. Materials and Methods

### 2.1. Identification of Coding Sequences of Core Genes of L. dispar TLR Pathway

TLR pathway core genes (*TLRs*, *MyD88*, *IRAK1*, *IRAK4*, *JNK* and *AP-1*) were identified from a previously constructed *L. dispar* transcriptome [15]. Open reading frames (ORFs) and deduced amino acid (aa) sequences were determined using NCBI ORF finder (https://www.ncbi.nlm.nih.gov/orffinder/, accessed on 6 March 2021). The domain architectures of identified genes were characterized using the SMART program (http://smart.embl-heidelberg.de/, accessed on 6 March 2021). The ExPASy server (https://web.expasy.org/compute_pi/, accessed on 7 March 2021) was used to calculate the molecular weights (Mw) and isoelectric point (pI) values.

### 2.2. Primary and 3-Dimensional Structural Analysis

Amino acid sequence of the TIR domains in eleven LdTLRs and LdMyD88 were aligned using Clustal Omega [16]. The MEME program version 5.3.3 (https://meme-suite.org/meme/tools/meme, accessed on 12 April 2021) was used to generate the sequence logos. Three-Dimensional (3D) structures of the TIR domians were obtained by homology modeling (https://swissmodel.expasy.org/, accessed on 14 April 2021). PyMOL software version 2.1 was used to perform the structural alignment and the calculation of the Root-mean-square deviations (RMSD). All 3D structural figures were visualized using PyMOL software.

### 2.3. Phylogenetic Analysis of L. dispar TLRs

The amino acid sequences of 11 *L. dispar* TLRs and 58 Toll/TLRs from another 10 insect species (Table S1) were aligned using the MAFFT method with the auto algorithm and BLOSUM62 scoring matrix [17]. The LG + R6 model was determined to be the best-fit model of amino acid evolution for insect TLRs using ModelFinder integrated in PhyloSuite version 1.2.2 with the default settings according to Bayesian information criterion (BIC) [18,19]. The maximum likelihood (ML) tree was then constructed using IQ-TREE under Ultrafast bootstrap with 5000 replicates, as well as the SH-aLRT test with 1000 replicates [20,21,22]. The tree was rooted by midpoint approach.

### 2.4. Insect Maintenance and Immune Challenge

Eggs of *L. dispar* and the artificial diet used to feed the animals were obtained from Chinese Academy of Forestry, Beijing, China. The *L. dispar* larvae were reared under the following conditions: temperature 26 ± 1 °C, relative humidity 60 ± 5% and a photoperiod of 16:8 h light (L): dark (D). LdMNPV occlusion bodies (OBs) were reproduced in fourth instar *L. dispar* larvae, and then purified by the steps of maceration, homogenate, filtration, centrifugation and sucrose gradient ultracentrifugation as described previously [23]. Newly molted third instar larvae were starved for 24 h and then purified suspension of LdMNPV OBs was fed by droplet feeding method [24] under the dosage of 2 × 10^6^ OBs/larva that has been screened [25]. The same volume of 10% sucrose solution was fed to control samples in the same way. Nine insect individuals treated with LdMNPV as well as nine larvae for mock-infection were collected at different days post infection (1, 2, 3, 4, 5 and 6 dpi). To minimize individual genetic differences, three larvae with same treatment were pooled together as one biological replicate. There were three biological replicates. All samples were snap frozen in liquid nitrogen and stored at −80 °C until further experimentation.

### 2.5. RNA Extraction and cDNA Synthesis

Total RNA was extracted from different samples using Trizol reagent (TianGen Biotech (Beijing, China) Co., Ltd. (Beijing, China)) according to the manufacturer’s instructions and subsequently treated with RNase-free DNase I (TianGen Biotech (Beijing) Co., Ltd. (Beijing, China)) to remove residual genomic DNA. The concentration of extracted RNA was measured by spectrophotometry and the quality was determined by 1% agarose denaturing gel electrophoresis. The synthesis of first strand cDNA was performed from each RNA sample (2 μg) using Transcriptor First Strand cDNA Synthesis Kit (Roche). The synthesized cDNA was stored at −20 °C until further analysis.

### 2.6. Quantitative Real-Time RT-PCR (qRT-PCR)

Expression levels of core genes of the TLR signaling pathway in this study were determined by qRT-PCR. The qRT-PCR assay was carried out with an LightCycler 480 instrument (Roche). The reaction mixture (20 μL) contained 3 μL of sterilized PCR-grade water, 5 μL of synthesized cDNA, 10 μL of Faststart essential DNA green master mix (Roche), and 1 μL of each primer (10 μM). The PCR procedure was implemented as follows: 95 °C for 10 min, followed by 45 cycles of 10 s at 95 °C, 10 s at 60 °C and 20 s at 72 °C. Finaly, a melting curve was generated for each sample to assess the specificity of the amplified products. For normalization purpose, the *Beta-tubulin* (*TUB*) and *Elongation factor 1 alpha* (*EF1-α*) were selected as the qRT-PCR internal control genes [26,27]. Target-specific primers were designed using the primer premier 5.0 software and provided in Table 1. The amplification efficiencies of the target and reference were approximately equal and ranged from 95% to 105%. All samples of three larvae mix were analyzed with three independent biological replicates. Each biological replicate includes three technical replicates. Expression levels of target genes were calculated using the 2^−ΔΔCt^ method [28].

### 2.7. Statistical Analysis

The data were presented using scatter dot plot with a horizontal line at median. Wilcoxon’s rank sum test (WRST) in SPSS 26.0 was used to compare statistical significance between two groups. *p* < 0.05 was considered to be statistically significant.

## 3. Results

### 3.1. Identification of the TLR Signaling Pathway Core Genes in L. dispar

To explore the potential antiviral roles of TLR pathway in *L. dispar*, eleven members of *TLR* family were identified from the *L. dispar* transcriptome database, namely *LdTLR1–11*. In addition, five downstream genes of the TLR-mediated signaling pathway were also found, and these genes were named *LdMyD88*, *LdIRAK1*, *LdIRAK4*, *LdJNK* and *LdAP-1*, separately. The ORF lengths of the 11 *TLR* genes ranged from 2010 bp (*LdTLR4*) to 4035 bp (*LdTLR10*), encoding proteins of 669 aa to 1344 aa in length (Table 2). The maximum and minimum molecular weight (MW) of LdTLRs were 153.11 kDa and 77.38 kDa, respectively (Table 2). The isoelectric points (pI) of all LdTLRs varied from 5.36 (LdTLR6) to 7.01 (LdTLR9) (Table 2). The detailed information of *LdMyD88*, *LdIRAK1*, *LdIRAK4*, *LdJNK* and *LdAP-1* and their coding proteins were listed in Table 2.

**Figure 1:**
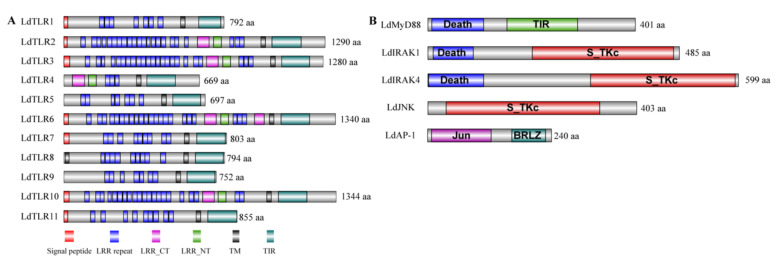
(**A**) The predicted protein domain architecture of the 11 Toll-like receptor genes from *L. dispar*. The various TLR genes contain numerous extracellular leucine-rich repeats (LRRs) domains, leucine-rich repeats C-terminal (LRR-CT), leucine-rich repeats N-terminal (LRR-NT), signal peptides, a transmembrane domain (TM) and toll-interleukin 1 receptor (TIR) domain. (**B**) Domain architecture of downstream TLR pathway components of *L. dispar* including LdMyD88, LdIRAK1, LdIRAK4, LdJNK and LdAP-1.

To better understand the biological functions of these core genes of TLR signaling pathway, the conserved protein domain architecture was analyzed and annotated. As shown in Figure 1A, all 11 LdTLRs contained an extracellular domain with 3–23 LRRs, a transmembrane region and a TIR domain. Additionally, except for LdTLR4, LdTLR5, LdTLR8 and LdTLR9, all LdTLRs were found to comprise a signal peptide. The conserved protein domains of LdMyD88, LdIRAK1, LdIRAK4, LdJNK and LdAP-1 were shown in Figure 1B. The protein encoded by LdMyD88 is composed of two protein interaction domains, a death domain and a TIR domain. The putative LdIRAK1 and LdIRAK4 proteins contain two domains, a death domain and a Serine/Threonine protein kinase, catalytic domain (S_TKc). The putative LdJNK protein possesses a S_TKc domain. The putative LdAP-1 protein contains two domains, a Jun-like transcription factor and a basic region leucin zipper (BRLZ).

### 3.2. Structural Analysis of TIR Domain of TLRs and MyD88 in L. dispar

**Figure 2:**
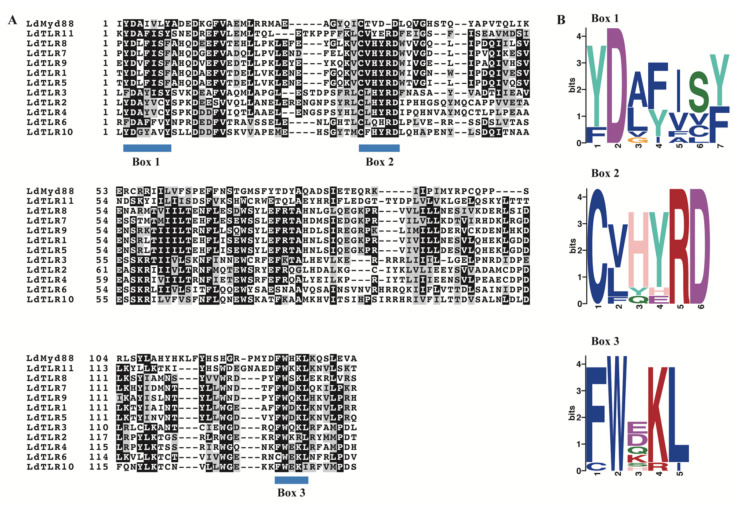
(**A**) Sequence alignment of TIR domains in 11 LdTLRs and LdMyD88. Blue bars indicate conserved motifs of TIR domains. (**B**) Sequence logos of Box 1–3 in the TIR domains of 11 LdTLRs and LdMyD88. Numbers on the horizontal axis represent the sequence positions in the motifs and the vertical axis represents the information content measured in bits.

The TLR family is a typical transmembrane receptor and composed by two major regions, one is the extracellular LRR domain and another is the intracellular TIR domain which is about 140 residues and essential for intracellular signal transduction. Besides TLRs, other immune-related proteins also contain TIR domains, such as MyD88 which is a cytoplasmic adaptor molecule of the TLR. The TIR domains were characterized by three highly conserved regions (boxes) [29,30]. The amino acid sequences alignment of TIR domains in LdTLRs and LdMyD88 was performed. Similar to other proteins that contain TIR domains, three conserved and functional motifs (Box 1, Box 2 and Box 3) were identified (Figure 2A). Furthermore, sequence logos analyses were performed using the MEME program. Box 1 was located at the N terminal of TIR domain containing a highly conserved Aspartate (D). Three highly conserved Cysteine (C), Arginine (R) and Aspartate (D) residues were observed in Box 2. Box 3 located at the C terminal of the TIR domains contained a highly conserved Tryptophan (W) (Figure 2B).

**Figure 3:**
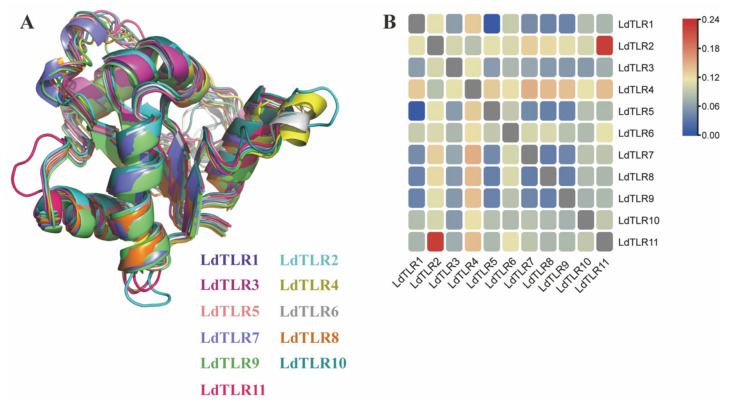
(**A**) Structural alignment of TIR domains of 11 LdTLRs, the colors correspond to different proteins. (**B**) RMSD matrix of TIR domains of 11 LdTLRs.

Additionally, the 3D structures of TIR domains of LdTLRs were obtained. The 3D structures of these TIR domains indicated that they possessed similar patterns in components arrangement, centrally located 4 parallel β sheets surrounded by 5–7 α helices (Figure S1). The 3D structural alignment of the TIR domain of LdTLRs showed that these TIR domains were similar in spatial conformation (Figure 3A), and the root-mean-square deviations (RMSD) ranged from 0.005 to 0.225 (Figure 3B), which suggested TIR domains of LdTLRs were structurally conserved during evolution.

### 3.3. Phylogenetic Analysis of LdTLRs

**Figure 4:**
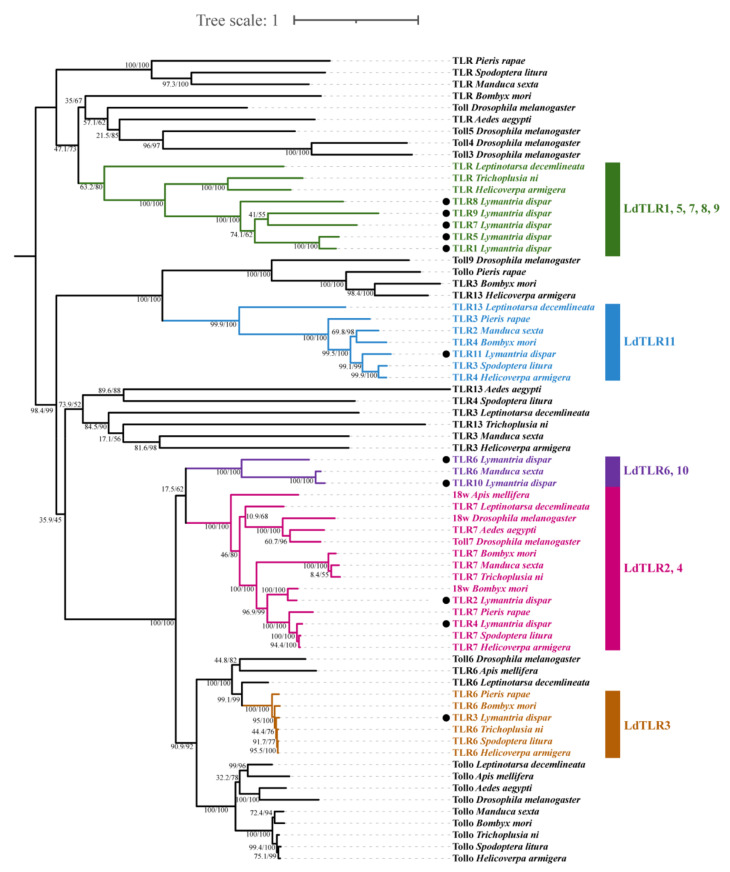
A phylogenetic tree of insect TLR proteins from the related species. Bootstrap support values and SH-aLRT values are indicated on branches. The tree was rooted by midpoint approach. Eleven LdTLRs were marked by black dots. Five clusters including LdTLRs are marked by different colors.

A phylogenetic tree was constructed based on the amino acid sequences of 11 TLRs from *L. dispar* and 58 Toll/TLRs from another 10 insects (Figure 4). The results revealed that *L. dispar* TLRs were placed into five clusters. LdTLR1, 5, 7, 8 and 9 were grouped into a monophyletic group and positioned as a sister taxon to the clade containing *Helicoverpa armigera* TLR and *Trichoplusia ni* TLR. LdTLR11 was placed as a sister taxon to the clade composed of *Spodoptera litura* TLR3 and *H. armigera* TLR4. LdTLR6, *Manduca sexta* TLR6 and LdTLR10 were grouped into a monophyletic group and LdTLR10 was placed on the same branch with *M. sexta* TLR6 with high support value. LdTLR2 is the homolog of *Bombyx mori* 18w. LdTLR4 was placed on the same branch with *S. litura* TLR7 and *H. armigera* TLR7. It seems that LdTLR3, *Pieris rapae* TLR6, *B. mori* TLR6, *T. ni* TLR6, *S. litura* TLR6 and *H. armigera* TLR6 possess common ancestor.

### 3.4. Expression Analysis of L. dispar Toll-Like Receptors upon LdMNPV Infection

**Figure 5:**
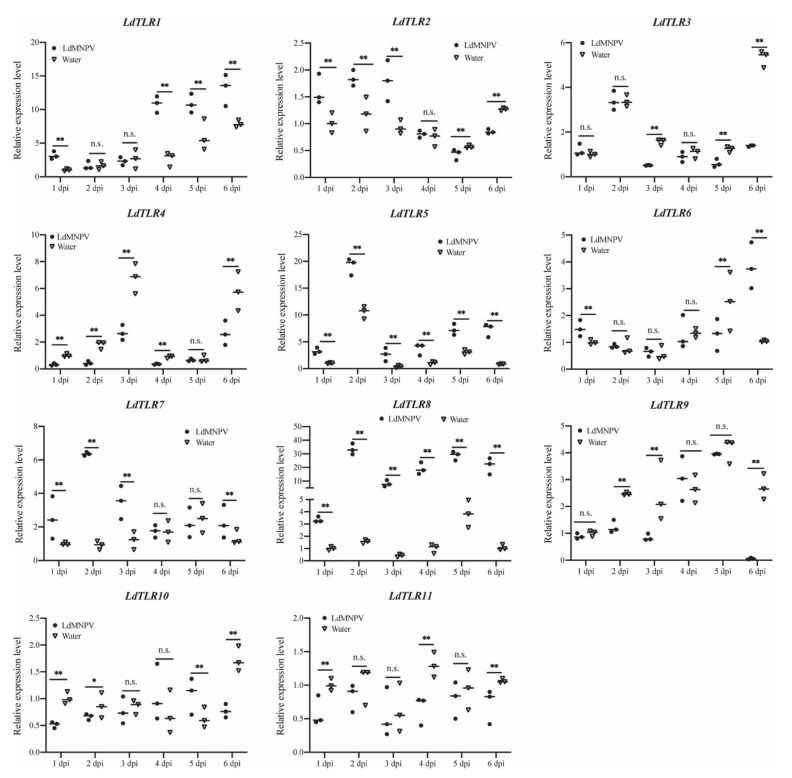
Relative mRNA expression levels of *L. dispar* Toll-like receptors upon LdMNPV infection. The horizontal line represents the median. Statistically significant differences are indicated with * (*p* < 0.05) and ** (*p* < 0.01).

Increasing evidence has demonstrated that TLRs elicit potent post-immune responses upon pathogenic stress. To explore the putative innate immune roles of *LdTLRs* in response to LdMNPV infection, quantitative RT-PCR-based expression profiling was conducted. The temporal expression profiles of these genes were observed at different days after LdMNPV infection and compared with uninfected samples. Significantly up-regulated mRNAs of *LdTLR1*, *LdTLR2*, *LdTLR5*, *LdTLR6*, *LdTLR7* and *LdTLR8* were observed after LdMNPV infection (Figure 5). In detail, *LdTLR1* mRNA was significantly up-regulated at one (3.13-fold), four (4.05-fold), five (1.80-fold) and six (1.67-fold) days post infection (dpi). *LdTLR2* mRNA was significantly up-regulated at one (1.59-fold), two (1.56-fold) and three (1.91-fold) dpi and remarkably down-regulated at five (1.31-fold) and six (1.49-fold) dpi. The expression levels of *LdTLR5* and *LdTLR8* remained up-regulated during the period of LdMNPV challenge from one to six days. After LdMNPV infection, the fold change of *LdTLR5* mRNA was highest at six dpi (nine-fold), and the transcription level of *LdTLR8* was highest induced at two dpi (21.26-fold). The *LdTLR6* was significantly up-regulated at one (1.51-fold) and six (3.69-fold) dpi and down-regulated at five (1.97-fold) dpi. Significantly up-regulated *LdTLR7* was detected at one (2.52-fold), two (6.93-fold), three (2.94-fold) and six (1.69-fold) dpi.

The expression levels of *LdTLR3*, *LdTLR4*, *LdTLR9*, *LdTLR10*, and *LdTLR11* were significantly suppressed upon LdMNPV infection (Figure 5). The expression level of *LdTLR3* was not changed at one, two and four dpi and significantly down-regulated at three (3.04-fold), five (2.04-fold) and six (3.84-fold) dpi. Except for five dpi, the expression level of *LdTLR4* was significantly suppressed at other days post infection and the fold changes were 2.99-, 4.18-, 2.53-, 2.50- and 2.18-fold separately. *LdTLR9* mRNA was significantly down-regulated at two (2.01-fold), three (2.96) and six (45,42-fold) dpi. *LdTLR10* mRNA was significantly down-regulated at one (1.96-fold), two (1.30-fold), and six (2.24-fold) dpi and remarkedly induced at five (1.68-fold) dpi. The expression level of *LdTLR11* was significantly down-regulated at one (1.70-fold), four (1.99-fold) and six (1.55-fold) dpi. These results imply that some of *LdTLRs* may be associated with antiviral response against LdMNPV infection but it requires further characterization.

### 3.5. Activation of Downstream TLR Pathway Factors of L. dispar in Response to LdMNPV Challenge

**Figure 6:**
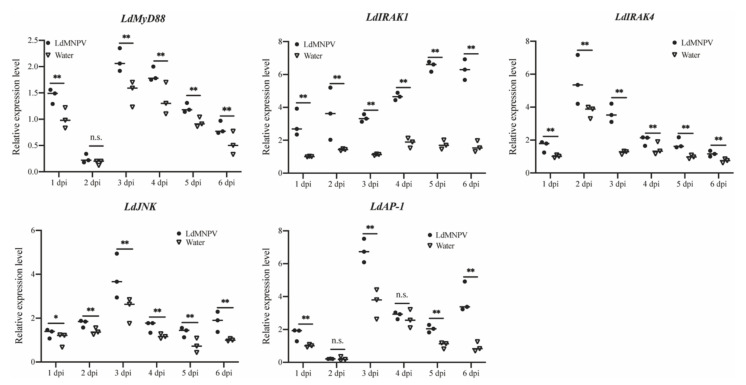
Relative mRNA expression levels of five downstream genes of TLR-mediated signaling pathway in *L. dispar* after LdMNPV infection. The horizontal line represents the median. Statistically significant differences are indicated with * (*p* < 0.05) and ** (*p* < 0.01).

It is well-known that stimulated TLRs can ultimately activate transcription factors including NF-κB and AP-1 [31]. Hence, mRNA expression patterns of some identified downstream TLR signaling pathway components including *LdMyD88*, *LdIRAK1*, *LdIRAK4*, *LdJNK* and *LdAP-1* in *L. dispar* were analyzed. As shown in Figure 6, expression level of five downstream TLR pathway factors were significantly induced after LdMNPV infection. The significantly enhanced expression of *LdMyD88* was observed at one (1.43-fold), three (1.41-fold), four (1.35-fold), five (1.29-fold) and six days (1.55-fold) after LdMNPV infection. The expression levels of *LdIRAK1*, *LdIRAK4* and *LdJNK* remained up-regulated during the period of LdMNPV challenge from one to six days and were highest induced at six (3.93-fold), three (2.90-fold) and six (1.86-fold) dpi, respectively. *LdAP-1* mRNA was significantly up-regulated at one (1.72-fold), three (1.88-fold), five (1.97) and six dpi (4.15-fold) after LdMNPV infection. Above results indicated that TLR signaling pathway of *L. dispar* was activated after LdMNPV invasion.

## 4. Discussion

Innate immunity provides the first defense line for most animals. The TLR superfamily recognizes various conserved microbial components such as cell wall components, double- and single-stranded RNA and unmethylated CpG DNA [32,33] and have been found in species ranging from nematodes to primates [34]. Nevertheless, few TLRs have been described in *L. dispar* and particularly their immune function role in response to baculovirus challenge was still obscure. In the present study, 11 *TLR* genes from *L. dispar* were identified. These TLRs contains typical TLR family domains, an extracellular domain with 3–23 LRRs, transmembrane region, and cytoplasmic TIR domain. The LRR is linked to ligand recognitions for the subsequent signal transduction. The number and arrangement of LRRs that exist in the extracellular region vary based on the type of TLR and the host species. For instance, the number of LRRs ranges from 1 to 24 in invertebrate [35]. The exact reason for the variation in the number of LRRs is still unclear, but it may be related to ligand affinity. The variation in the number of LRR domains indicate that LdTLRs may possess different mode of ligand recognitions.

The TLR-mediated signal pathway can trigger a cascade reaction and ultimately result in the expression of immune effector genes, which plays a crucial role in the process of immune defense. Therefore, some downstream TLR signaling pathway components in *L. dispar* including LdMyD88, LdIRAK1, LdIRAK4, LdJNK and LdAP-1 were also identified. LdMyD88 contains a death domain and a TIR domain. LdIRAK1 and LdIRAK4 share common structural features with a death domain and a S_TKc domain. The S_TKc domain is also present in LdJNK, a member of mitogen-activated protein kinases (MAPK) family. LdAP-1 contains two domains, a Jun-like transcription factor and a BRLZ. MyD88 allows recruitment of IRAK4, IRAK1 and/or IRAK2, a small family of serine/threonine kinases, to form a complex via a death domain interaction [36,37]. Subsequently, activated IRAKs recruit TNF receptor-associated-factor-6 (TRAF6) to trigger downstream molecule transforming growth factor (TGF)-β-activated kinase (TAK) 1, which then activate downstream signals, such as nuclear factor-κB (NF-κB) and MAPK [38,39]. Members of the MAPK family leads to phosphorylation of transcription factor AP-1 which then induces the production of inflammatory cytokines [31]. Besides, MyD88 is also related to interferon-regulatory factor 7 (IRF7). IRAK1 is phosphorylated by IRAK4 recruited by MyD88 and then IRF7 is phosphorylated by activated IRAK1 to induce the production of type I interferons which are key cytokines to respond to viruses [40].

The TIR domain which is the intracellular component of TLRs interacts with some downstream TIR-containing adapter proteins, such as MyD88 [41]. Sequence alignments of TIR domains among LdTLRs and LdMyD88 showed these TIR domains contained three functional motifs which were named Box 1–3. Box 1 and Box 2 are associated with initiating downstream signaling cascades [42] while Box 3 is involved in maintaining receptor localization [43]. These three highly conserved motifs enabled the functional congruency of the TIR domains in varied LdTLRs. Structural alignments of TIR domains among LdTLRs showed that they possessed similar structural components and spatial arrangements, which further demonstrated the functional conservation of the TIR domains of LdTLRs.

A phylogenetic tree was constructed based on amino acid sequences of 11 *L. dispar* TLRs and 58 Toll/TLRs from other 10 insect species. The LdTLRs were placed into five clusters. LdTLR1, 5, 7, 8 and 9 clustered into a monophyletic group which was positioned as a sister taxon to the clade containing *T. ni* TLR and *H. armigera* TLR, implying that LdTLR1, 5, 7, 8 and 9 may play special roles in the innate immunity of *L. dispar.*

To further understand the role of TLR signaling pathway in the immune response, induction pattern of core genes of this pathway in response to LdMNPV was analyzed. The results indicated that expression levels of *LdTLR1*, *LdTLR2*, *LdTLR5*, *LdTLR6*, *LdTLR7* and *LdTLR8* were highly induced by LdMNPV. Five downstream TLR signaling pathway components including *LdMyd88*, *LdIRAK1*, *LdIRAK4*, *LdJNK* and *LdAP-1* were all up-regulated and this tendency persisted until six dpi. It can be inferred from above results that TLR signaling pathway was activated and might be involved in the immune response against LdMNPV infection.

In conclusion, eleven TLRs and five downstream TLR pathway components of TLR signaling pathway in *L. dispar* were identified and analyzed. Our results in this study will lay the foundation for further understandings of innate immunity of the pest, and provide a new opportunity to understand mechanisms of invertebrate responses to viral infection.

## Figures and Tables

**Table 1:** Sequences of the primers for qRT-PCR.

Gene	Forward	Reverse
*LdTLR1*	5′ GTTGTAGTAGAGGGTTGTGCTTC 3′	5′ AGTATGGTAACTGTTGATGGTGA 3′
*LdTLR2*	5′ TTACGATTAGACGGCAATAGGCT 3′	5′AATAACGACACTTGCACGACCAC 3′
*LdTLR3*	5′ TAGAGGCGGAATCATCTCAGTTT 3′	5′ CGTCATTTCACAATCACAAGCGT 3′
*LdTLR4*	5′ GCGGCATAGAGTGAAACAA 3′	5′ ATGAGACAGCACCCAAGAA 3′
*LdTLR5*	5′ CATACAAGAAGGGAAGCCAAGAG 3′	5′ CCAAAAGTAAACATCTCCCCACA 3′
*LdTLR7*	5′ CCATAGACATCGCAGACCAA 3′	5′ AAGCACAGCGTATCACCGTA 3′
*LdTLR8*	5′ CTTTTCATTTCCTTCGCACA 3′	5′ CACCGACAACCCAATCTCTA 3′
*LdTLR9*	5′ CGGCTAAACTTACACTTCGC 3′	5′ TTTCACATCTTTCACCATCG 3′
*LdTLR11*	5′ AGTGCTACTGTCGTCTTCCC 3′	5′ GTTATTGCTTCCTCCTCCTA 3′
*LdTLR13*	5′ TAAATAACAAATACGGTGGCAAA 3′	5′ CTTCAAAATCACAACAGAAACAA 3′
*LdTLR14*	5′ AGCCTCATTCCTGTCACCTTCT 3′	5′ ATGTCACCGACCCGTTTCTTAT 3′
*Ld* *MyD88*	5′ GTGATACTAAGTGACGGACCTGA 3′	5′ATGACTCTACCTCGGAAGTTATG 3′
*LdIRAK1*	5′ GTTTCAAGAGTCTATGGCACCCG 3′	5′ TCTAAAGCTGAAAGTGTGGTCGC 3′
*LdIRAK4*	5′ ATGGCACCAGAAGGTTTATCAGG 3′	5′ ACCGCCCACAGTTACAATAGGAC 3′
*LdJNK*	5′ CGCATTCACGCCACAGAAGAGTC 3′	5′ TGGCACAGGTTTGCGTCCATTAG 3′
*LdAP-1*	5′ ACAGTGCCCAGTGCCTCCAGTA 3′	5′ CGATTCCTTTGCCTTTTGCGTT 3′
*EF1-α*	5′ TTTGCCTTCCTTGCGCTCAACA 3′	5′ TGTAAAGCAGCTGATCGTGGGT 3′
*TUB*	5′ AATGCAAGAAAGCCTTGCGCCT 3′	5′ ATGAAGGAGGTCGACGAGCAAA 3′

**Table 2:** Characterization of identified core genes of TLR signaling pathway in *L. dispar*.

Gene	ORF Length (bp)	Protein Sequence (aa)	Mw (kDa)	pI
*LdTLR1*	2379	792	91.03	6.75
*LdTLR2*	3873	1290	146.86	6.12
*LdTLR3*	3843	1280	146.41	5.95
*LdTLR4*	2010	669	77.38	6.11
*LdTLR5*	2094	697	80.29	6.00
*LdTLR6*	4023	1340	152.33	5.36
*LdTLR7*	2424	803	92.03	6.09
*LdTLR8*	2385	794	91.63	5.89
*LdTLR9*	2259	752	88.28	7.01
*LdTLR10*	4035	1344	153.11	6.03
*LdTLR11*	2568	855	99.24	6.59
*LdMyD88*	1206	401	45.61	6.43
*LdIRAK1*	1458	485	55.55	8.84
*LdIRAK4*	1800	599	67.33	6.63
*LdJNK*	1212	403	45.88	6.46
*LdAP-1*	723	240	27.17	9.3

## Data Availability

No new data were created or analyzed in this study. Data sharing is not applicable to this article.

## Supplementary Materials

The following are available online at https://www.mdpi.com/article/10.3390/insects12090827/s1, Figure S1: 3D structures of TIR domains in 11 LdTLRs, Table S1: genBank accession numbers of TLRs form other insect species.
