# Supplementary material for: Identification of Core Genes of Toll-like Receptor Pathway from Lymantria dispar and Induced Expression upon Immune Stimulant
Source: Insects. 2021 Sep 14;12(9):827. doi: 10.3390/insects12090827 (PMC8469855; doi:10.3390/insects12090827)
Supplement: Supplementary file 1 [file insects-12-00827-s001.zip › insects-1300464-supplementary.pdf]

Table S1. GenBank accession numbers of TLRs form other insect species.

Toll/TLR	Species	GenBank accession No.
TLR	*Helicoverpa armigera*	XP_021197312.1
TLR3	*Helicoverpa armigera*	XP_021187138.1
TLR4	*Helicoverpa armigera*	XP_021190980.1
Tollo	*Helicoverpa armigera*	XP_021181728.1
TLR6	*Helicoverpa armigera*	XP_021181725.1
TLR7	*Helicoverpa armigera*	XP_021193405.1
TLR13	*Helicoverpa armigera*	XP_021199918.1
TLR	*Bombyx mori*	XP_004925675.1
18w	*Bombyx mori*	NP_001116821.1
TLR3	*Bombyx mori*	XP_012549875.2
TLR4	*Bombyx mori*	XP_012546905.2
TLR6	*Bombyx mori*	XP_012553339.2
TLR7	*Bombyx mori*	XP_004921732.2
Tollo	*Bombyx mori*	XP_004921742.1
TLR	*Trichoplusia ni*	XP_026724543.1
TLR6	*Trichoplusia ni*	XP_026733863.1
TLR7	*Trichoplusia ni*	XP_026733855.1
Tollo	*Trichoplusia ni*	XP_026733502.1
TLR13	*Trichoplusia ni*	XP_026737397.1
18w	*Apis mellifera*	NP_001013379.1
TLR6	*Apis mellifera*	XP_393712.2
Tollo	*Apis mellifera*	XP_393713.3
Toll	*Drosophila melanogaster*	AAA28941.1
18w	*Drosophila melanogaster*	AAF57509.1
Toll3	*Drosophila melanogaster*	AAF54021.3
Toll4	*Drosophila melanogaster*	AAF52747.3
Toll5	*Drosophila melanogaster*	AAF53306.1
Toll6	*Drosophila melanogaster*	AAF49645.1
Toll7	*Drosophila melanogaster*	AAF57514.1
Tollo	*Drosophila melanogaster*	AAF49650.1
Toll9	*Drosophila melanogaster*	AAF51581.1
TLR	*Manduca sexta*	XP_030022832.2
TLR2	*Manduca sexta*	XP_030022594.2
TLR3	*Manduca sexta*	XP_030032678.1
TLR6	*Manduca sexta*	XP_030022524.1
TLR7	*Manduca sexta*	XP_030022522.1
Tollo	*Manduca sexta*	XP_030027852.2
TLR	*Spodoptera litura*	XP_022826408.1
TLR3	*Spodoptera litura*	XP_022823165.1
TLR4	*Spodoptera litura*	XP_022821797.1
TLR6	*Spodoptera litura*	XP_022828061.1
TLR7	*Spodoptera litura*	XP_022828080.1
Tollo	*Spodoptera litura*	XP_022828179.1
TLR	*Pieris rapae*	XP_022126205.1
Tollo	*Pieris rapae*	XP_022117126.1
TLR3	*Pieris rapae*	XP_022116081.1
TLR6	*Pieris rapae*	XP_022121761.1
TLR7	*Pieris rapae*	XP_022129543.1
TLR	*Aedes aegypti*	XP_021713350.1
TLR7	*Aedes aegypti*	XP_001655730.1
Tollo	*Aedes aegypti*	XP_001649813.1
TLR13	*Aedes aegypti*	XP_021701630.1
TLR	*Leptinotarsa decemlineata*	XP_023026371.1
TLR3	*Leptinotarsa decemlineata*	XP_023021962
Tollo	*Leptinotarsa decemlineata*	XP_023024566.1
TLR6	*Leptinotarsa decemlineata*	XP_023013197.1
TLR7	*Leptinotarsa decemlineata*	XP_023022264
TLR13	*Leptinotarsa decemlineata*	XP_023020393.1

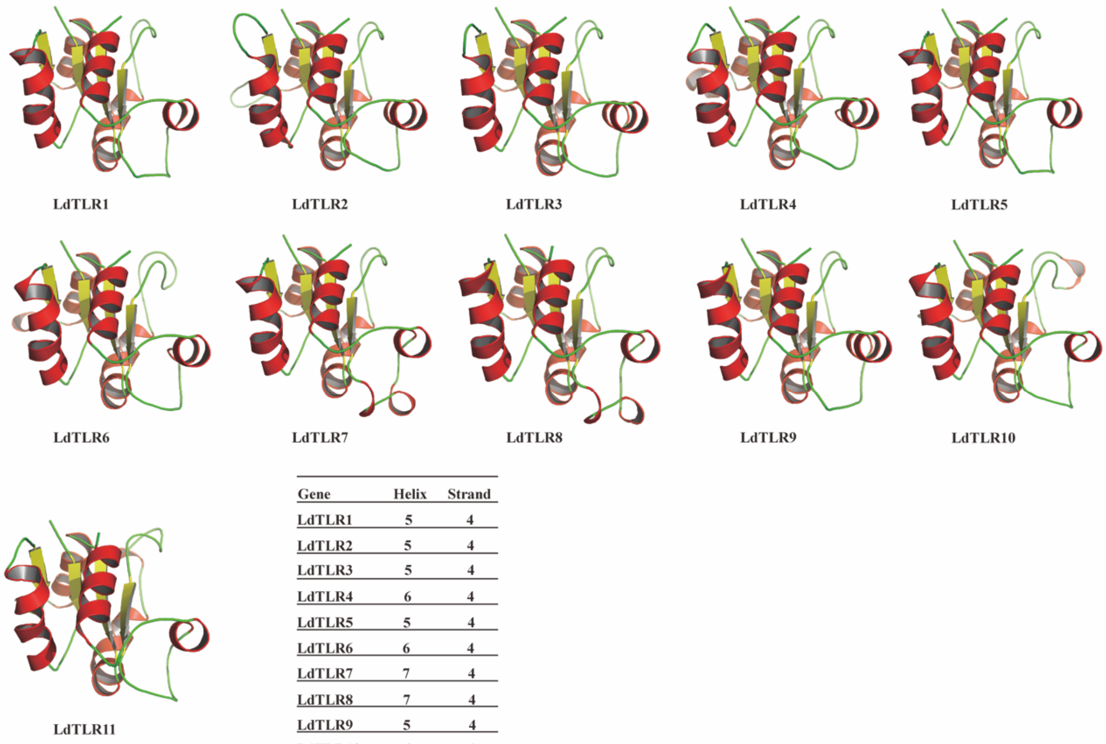

Gene	Helix	Strand
LdTLR1	5	4
LdTLR2	5	4
LdTLR3	5	4
LdTLR4	6	4
LdTLR5	5	4
LdTLR6	6	4
LdTLR7	7	4
LdTLR8	7	4
LdTLR9	5	4
LdTLR10	6	4
LdTLR11	6	4

Figure S1. 3D structures of TIR domains in 11 LdTLRs.
